# Association between changes in pancreatic morphology and vascular complications in subjects with type 2 diabetes mellitus: a retrospective study

**DOI:** 10.1038/s41598-022-21688-1

**Published:** 2022-10-13

**Authors:** Yuichiro Iwamoto, Tomohiko Kimura, Fuminori Tatsumi, Toshitomo Sugisaki, Masato Kubo, Erina Nakao, Kazunori Dan, Ryo Wamata, Hideyuki Iwamoto, Kaio Takahashi, Junpei Sanada, Yoshiro Fushimi, Yukino Katakura, Masashi Shimoda, Shuhei Nakanishi, Tomoatsu Mune, Kohei Kaku, Hideaki Kaneto

**Affiliations:** Department of Diabetes, Endocrinology and Metabolism, Kawasaki Medical School, 577 Matsushima, Kurashiki, 701-0192 Japan

**Keywords:** Endocrinology, Medical research

## Abstract

Decreased pancreatic volume, increased pancreatic fat mass, and serrated pancreatic margins are characteristic morphological changes of the pancreas in subjects with type 2 diabetes mellitus. This retrospective study aimed to clarify the clinical significance of pancreatic morphological changes in subjects with type 2 diabetes mellitus who underwent abdominal magnetic resonance imaging. The mean age and HbA1c value were 59.1 ± 16.3 years old and 8.9 ± 2.3%, respectively. Pancreatic body mass corrected for body surface area (BSA) in subjects with diabetes mellitus was lower compared to those in normal glucose tolerance (49.4 ± 15.3 cm^3^ vs. 60.9 ± 7.8 cm^3^), although it did not reach a statistic significance. There was a negative correlation between BSA-corrected pancreatic volume and age, duration of diabetes, glycoalbumin, mean and max IMT, and there was a positive correlation between BSA-corrected pancreatic volume and HOMA2-β. Serration of the pancreatic limbus was more often observed in subjects with diabetes mellitus compared to those in normal glucose tolerance (74.1% vs. 14.3%). Subjects with serrated changes were older and had higher HbA1c, and visceral fat area was significantly larger in subjects with serrated changes. BSA-corrected pancreatic volume in subjects with serrated changes was significantly smaller, and mean IMT was significantly thicker in subjects with serrulation. Furthermore, advanced diabetic retinopathy and diabetic nephropathy were more often observed in subjects with serrated changes. Taken together, decreased BSA-corrected pancreatic volume and serrated changes were associated with the progression of vascular complications in subjects with type 2 diabetes mellitus.

## Introduction

Type 2 diabetes mellitus is a disease that causes β-cell dysfunction due to genetic and environmental factors; it is estimated that β-cell function is reduced by 50% at the onset of type 2 diabetes mellitus^1^. Previous studies using ultrasonography and computed tomography (CT) to evaluate pancreatic volume have shown a 7–22% decrease in pancreatic volume in subjects with type 2 diabetes compared to subjects without glucose intolerance^2^^,^^3^. Decreased β-cell counts and fat deposition and fibrosis in the pancreas have been postulated as reasons for the decreased pancreatic volume in type 2 diabetes^4^. Magnetic resonance imaging (MRI) has superior spatial resolution compared to CT, and it is more sensitive to the intra-abdominal fat than CT. The use of MRI for the safe evaluation of pancreatic morphology has led to studies in subjects with type 2 diabetes mellitus, and the results of these studies showed that the pancreas is a characteristic pancreatic organ in type 2 diabetes mellitus^5^. Imaging findings included decreased pancreatic volume, increased pancreatic fat content, and serrated pancreatic limbus. There was a significant correlation between pancreatic volume and homeostatic model assessment β-cell function (HOMA-β). Previous studies have suggested that morphological changes in the pancreas in type 2 diabetes mellitus may have some effect on endogenous insulin secretion^6^. Although there is concern about the possible association between morphological changes in the pancreas and diabetes-related complications, there have been no reports examining the relationship between diabetes-related complications and changes in pancreatic morphology. This study aims to clarify the clinical significance of pancreatic morphological changes on diabetes-related complications and pathological progression in subjects with type 2 diabetes who underwent abdominal MRI.

## Materials and methods

### Study subjects

This retrospective study conducted at the Department of Diabetes, Metabolism, and Endocrinology, Kawasaki Medical School, Japan, included adult subjects aged 20 years or older who underwent abdominal MRI at our department between December 1, 2018, and October 31, 2021. The study was approved by the Ethics Committee of Kawasaki Medical School and its affiliated hospitals (No. 5500-00).

A total of 95 adult subjects were screened; those with type 1 diabetes mellitus (n = 4), cases complicated with malignancy (n = 20), those taking steroids (n = 3), cases in which insulin secretory capacity was not evaluated (n = 1), and cases in which pancreatic volume could not be calculated from the imaged images (n = 2) were excluded from the study. A total of 65 subjects, 58 diabetic and 7 non-diabetic, were finally included in the analysis. In non-diabetic patients who underwent MRI, 6 cases had non-functioning adrenal adenomas and 1 case experienced hypoglycemia.

### Methods

Pancreatic volume measurements and serrated changes of the pancreatic limbus were evaluated by two physicians trained by a radiologist and a radiology technologist. The pancreas was reconstructed into a 3D image using SYNAPSE VINCENT (FUJIFILM, Tokyo, Japan) from 3 to 5 mm thick T1 fat suppression images obtained by MRI. Pancreatic volume was corrected for body surface area (BSA), which was calculated using the formula: body weight 0.425 × height 0.725 × 0.007184^7^. The relationship between the obtained changes in pancreatic volume and pancreatic limbus and diabetes-related parameters at MRI imaging, physical examination, smoking history, alcohol consumption history, and carotid ultrasound was evaluated. Carotid IMT was assessed by an ultrasound technician with ultrasonographic equipment, Aplio series (CANON MEDICAL SYSTEMS, Tochigi, Japan). The carotid IMT was evaluated in the common carotid artery wall 10 mm centrally from the carotid sinus. 2 IMT points were measured within a 10 mm area, and the mean value was defined as mean IMT. The maximum diameter in the same measurement range was defined as max IMT. Data about diabetic retinopathy and nephropathy evaluated within 1 year before and after the MRI were included in the analysis. The Davis classification was used to evaluate diabetic retinopathy, which was grouped into non-diabetic retinopathy (NDR), simple diabetic retinopathy (SDR), pre-proliferative retinopathy (PPDR), and proliferative diabetic retinopathy (PDR). Diabetic nephropathy was evaluated by dividing the patients into 5 stages based on eGFR and albuminuria: Stage 1 was defined as eGFR > 30 mL/min/1.73 m^2^ and urinary albumin < 30 mg/gCr; Stage 2 was defined as eGFR > 30 mL/min/1.73 m^2^ and urinary albumin Stage 3: eGFR ≥ 30 mL/min/1.73 m^2^ and urinary albumin ≥ 300 mg/gCr, Stage 4: eGFR < 30 mL/min/1.73 m^2^, and Stage 5: patients on dialysis therapy. Urinary albumin was natural logarithmized when calculating the correlation coefficient with pancreatic volume. To assess endogenous insulin secretory capacity, HOMA2-β was calculated using the HOMA2 Calculator for Mac OS X Catalina and Linux (Diabetes Trials Unit, Oxford, England). Application program interface was downloaded from http://www.dtu.ox.ac.uk/homacalculator/index.php. All methods were carried out in accordance with relevant guidelines and regulations.

### Statistical analysis

Clinical characteristics of the subjects used in the analysis were age, laboratory findings at MRI imaging, endogenous insulin secretory capacity, and carotid ultrasound. Student's t-test was used to compare normal glucose tolerance cases with diabetic cases, and correlation analysis and multiple regression analysis were used to evaluate pancreatic volume corrected for BSA and each parameter. The changes in the pancreatic limbus and each parameter were evaluated using the chi-square test. Multiple regression analysis was performed to evaluate the impact of BSA-corrected pancreatic volume on HOMA2-β and mean IMT. The objective variables were HOMA2-β and mean IMT, and the explanatory variables were as follows: (model 1) age, sex, BMI, duration of type 2 diabetes, HbA1c, and BSA-corrected pancreatic volume; (model 2) age, sex, systolic blood pressure, LDL-cholesterol, HbA1c, and BSA-corrected pancreatic volume; (model 3) age, sex, BMI, Brinkmann’s index, HbA1c, and BSA-corrected pancreatic volume. Statistical software used were Excel Statistics for Mac version 16.54 (Social Research Information, Tokyo, Japan) and JMP version 16.0.1 (SAS Institute Inc.).

### Informed consent

Informed consent was obtained from all subjects in this study.

## Results

### Clinical characteristics

The clinical characteristics of the subjects with type 2 diabetes are shown in Table 1. The mean age was 59.1 ± 16.3 years, and 72.4% of the participants were male. HbA1c (National Glycohemoglobin Standardization Program [NGSP]) was 8.9 ± 2.3%. Duration of diabetes mellitus was 11.9 ± 9.1 years, which included many subjects with a relatively long history of diabetes mellitus. The prevalence of diabetic retinopathy was 24.1% (10.3% for SDR and 6.9% for PPDR and PDR, respectively). As for diabetic nephropathy, 14 patients (24.1%) had Stage 2, 8 (3.4%) had Stage 3, and 10 (17.2%) had Stage 4. The percentages of patients who had ever been diagnosed with dyslipidemia or hypertension were 89.5% and 77.2%, respectively.

**Table 1:** Various parameters in subjects with type 2 diabetes mellitus in this study (n = 58).

Parameters		Parameters	
Male/female	42/16	Immunoreactive insulin (μU/L)	10.2 ± 8.5
Age (years)	59.1 ± 16.3	C-peptide (ng/mL)	3.0 ± 2.0
Duration of diabetes (years)	11.9 ± 9.1	LDL-cholesterol (mg/dL)	106.0 ± 35.6
Body weight (kg)	75.3 ± 18.8	HDL-cholesterol (mg/dL)	43.7 ± 13.3
BMI (kg/m^2^)	27.6 ± 5.6	Triglyceride (mg/dL)	205.4 ± 178.8
Number of diabetic retinopathy		AST (U/L)	30.0 ± 22.5
Non-diabetic retinopathy	43 (74.1%)	ALT (U/L)	33.1 ± 30.5
Simple diabetic retinopathy	6 (10.3%)	Total protein (g/dL)	7.1 ± 0.4
Pre-proliferative diabetic retinopathy	4 (6.9%)	Albumin (g/dL)	4.1 ± 0.3
Proliferative diabetic retinopathy	4 (6.9%)	Creatinine (mg/dL)	1.08 ± 0.64
Number of diabetic nephropathy		Urea nitrogen (mg/dL)	20.0 ± 12.1
Stage 1/2/3	26/14/8	eGFR (mL/min/1.73m^2^)	66.6 ± 29.1
4/5	10/0	UA (mg/dL)	5.7 ± 1.4
Blood glucose levels (mg/dL)	151.2 ± 46.3	Amylase (U/L)	66.8 ± 31.9
HbA1c (%, NGSP)	8.9 ± 2.3	Pancreatic amylase (U/L)	26.7 ± 11.3
Glycoalbumin (%)	22.4 ± 8.2	CRP (mg/dL)	0.24 ± 11.3

Data presented as mean ± standard deviation. *BMI* body mass index, *LDL* cholesterol low-density lipoprotein cholesterol, *HDL* cholesterol high-density lipoprotein cholesterol, *eGFR* estimated glomerular filtration rate, *UA* uric acid, *CRP* C-reactive protein.

### Pancreatic volume and diabetes mellitus

**Figure 1:**
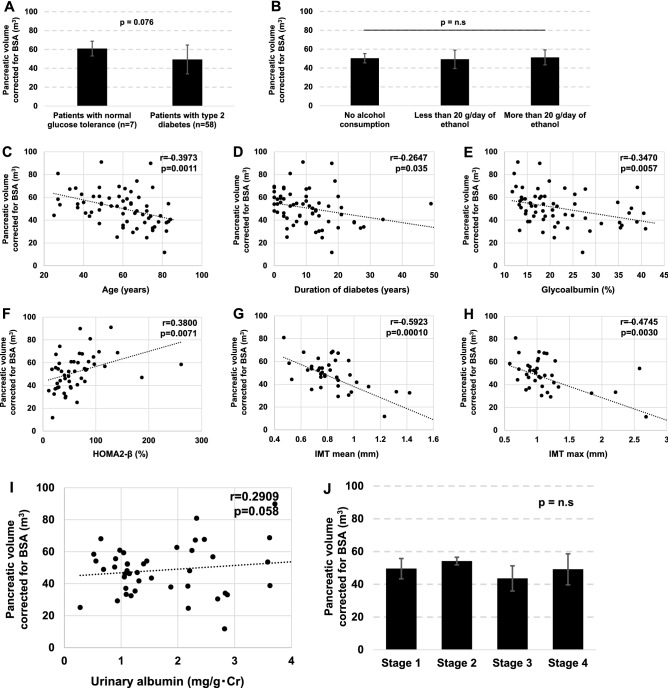
(**A**) Comparison of BSA-corrected pancreatic volume in subjects between with normal glucose tolerance and type 2 diabetes mellitus. (**B–J**) Correlation between BSA-corrected pancreatic volume and diabetes-related parameters. There was a negative correlation between BSA-corrected pancreatic volume and age, duration of diabetes, glycoalbumin, mean and max IMT, and there was a positive correlation between BSA-corrected pancreatic volume and HOMA2-β.

We evaluated how BSA-corrected pancreatic volume changes in subjects with diabetes mellitus (Fig. 1A). BSA-corrected pancreatic volume in subjects with normal glucose tolerance and diabetes mellitus were 60.9 ± 7.8 cm^3^ and 49.4 ± 15.3 cm^3^, respectively, although it did not reach a statistic significance. In the present study, pancreatic volume did not change when grouped by alcohol consumption (Fig. 1B). Next, we evaluated the correlation between BSA-corrected pancreatic volume and diabetes-related parameters. As shown in Figs. 1C–H, there was a negative correlation between BSA-corrected pancreatic volume and age, duration of diabetes, glycoalbumin, mean and max IMT, and there was a positive correlation between BSA-corrected pancreatic volume and HOMA2-β. The correlation between pancreatic volume and microvascular complications was evaluated (Fig. 1I,J). There was a positive correlation between albuminuria and pancreatic volume (*p* = 0.058). No significant correlation was found between progression of diabetic retinopathy and pancreatic volume (*p* > 0.05).

Multiple regression analysis was performed to evaluate factors affecting HOMA2-β and mean IMT. The results of the multiple regression analysis for HOMA2-β are shown in Table 2; BSA-corrected pancreatic volume was not an independent factor determining HOMA2-β (t = 1.03, *p* > 0.05). Table 3 shows the results of multiple regression analysis for mean IMT, which showed a significant correlation between BSA-corrected pancreatic volume and mean IMT (t = − 2.00, *p* < 0.05). Next, other atherosclerotic factors affecting mean IMT were included in the analysis. Model 2 included systolic blood pressure and LDL-cholesterol, which were independent factors affecting mean IMT (t = 2.52, *p* < 0.05). The t-value for BSA-corrected pancreatic volume in model 2 was − 1.74 (*p* = 0.061). In model 3, Brinkmann index was added to the analysis, and BSA-corrected pancreatic volume was an independent factor affecting mean IMT (t = − 2.06, *p* < 0.05).

**Table 2:** Multiple regression analysis about several factors influencing HOMA2-β levels.

Parameter	Standard β	*t* value	*p* value
Age (years)	− 0.324	− 1.77	n.s
Male (%)	0.042	0.33	n.s
BMI (kg/m^2^)	− 0.147	− 0.94	n.s
Duration of type 2 diabetes (years)	− 0.195	− 1.36	n.s
HbA1c (%)	− 0.371	− 2.69	< 0.05
BSA-corrected pancreatic volume (m^3^)	− 0.146	− 1.03	n.s

*BMI* body mass index, *BSA* body surface area.

**Table 3:** Multiple regression analysis about several factors influencing mean IMT.

	Standard β	t value	p value
**Parameter (model 1)**			
Age (years)	0.512	2.68	< 0.05
Male (%)	0.060	0.40	n.s
BMI (kg/m^2^)	0.005	0.76	n.s
Duration of type 2 diabetes (years)	− 0.018	− 0.12	n.s
HbA1c (%)	0.173	0.76	n.s
BSA-corrected pancreatic volume (m^3^)	− 0.326	− 2.00	< 0.05
**Parameter (model 2)**			
Age (years)	0.596	3.01	< 0.05
Male (%)	− 0.007	− 0.05	n.s
BMI (kg/m^2^)	0.062	0.33	n.s
systolic blood pressure (mmHg)	0.157	1.01	n.s
LDL-cholesterol (mg/dL)	0.337	2.52	< 0.05
BSA-corrected pancreatic volume (m^3^)	− 0.258	− 1.74	n.s
**Parameter (model 3)**			
Age (years)	0.526	2.76	< 0.05
Male (%)	0.073	0.53	n.s
BMI (kg/m^2^)	0.125	0.77	n.s
Brinkmann’s index	− 0.058	− 0.42	n.s
HbA1c (%)	0.177	1.35	n.s
BSA-corrected pancreatic volume (m^3^)	− 0.323	− 2.06	< 0.05

*BMI* body mass index, *BSA* body surface area, *LDL*-cholesterol low-density lipoprotein cholesterol.

### Serrated changes of the pancreatic margins

**Figure 2:**
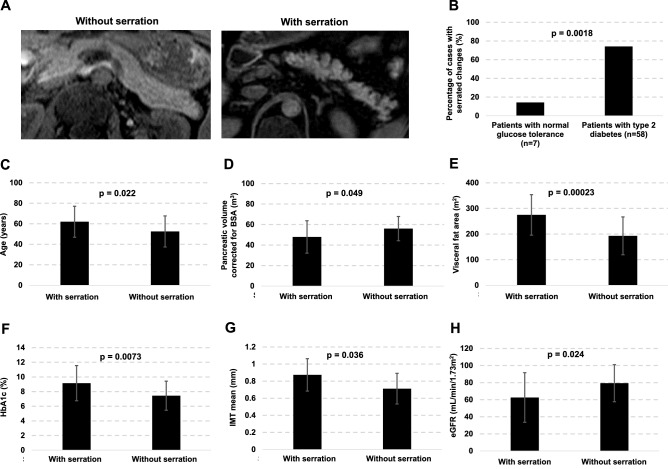
(**A**) Representative serrated changes of the pancreatic margins in subjects with type 2 diabetes mellitus. (**B**) Percentage of serrated pancreatic changes in subjects with normal glucose tolerance and type 2 diabetes mellitus. Percentage of serrated pancreatic changes was significantly higher in subjects with type 2 diabetes mellitus compared to those with normal glucose tolerance (*p* < 0.005). (**C–H**) Correlation between the presence of serrated pancreatic changes and various parameters. Subjects with serrated changes were older and had higher HbA1c (*p* < 0.05). Visceral fat area was significantly larger in subjects with serrated changes compared to those without them (*p* < 0.0005). BSA-corrected pancreatic volume in subjects with serrated changes was significantly smaller compared to those without them (*p* < 0.05), and mean IMT was significantly thicker in subjects with serrulation (*p* < 0.05).

**Figure 3:**
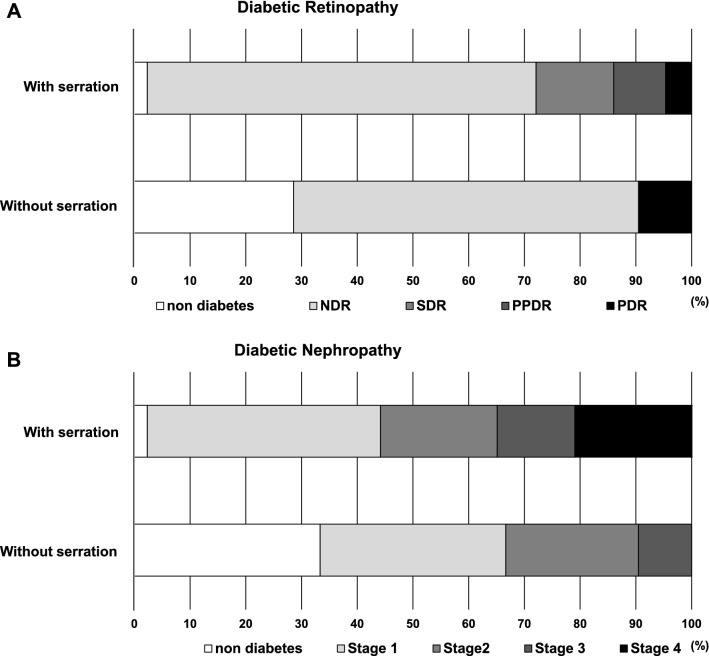
Possible association between the serrated pancreas and microvascular complications. Advanced diabetic retinopathy and nephropathy were more often observed in subjects with serrated changes compared to those without them (*p* < 0.05, *p* < 0.005).

The relationship between changes in the pancreatic limbus and various parameters was evaluated. The characteristic pancreatic limbus changes in diabetes mellitus is serrated changes (Fig. 2A). Serration of the pancreatic limbus was observed in 14.3% of subjects with normal glucose tolerance and 74.1% of those with diabetes mellitus (*p* < 0.005, Fig. 2B). Next, we evaluated the correlation between the presence of serrated pancreatic changes and various parameters (Figs. 2C–H). Subjects with serrated changes were older and had higher HbA1c. Visceral fat area was significantly larger in subjects with serrated changes (274.7 ± 79.2 cm^2^) compared to that in subjects without serrated changes (193.1 ± 73.8 cm^2^) (*p* < 0.0005). BSA-corrected pancreatic volume was 56.0 ± 11.8 cm^3^ in subjects without serrulation which was significantly larger compared to those with serrulation (47.9 ± 15.8 cm^3^) (*p* < 0.05), and mean IMT was significantly thicker in subjects with serrulation (*p* < 0.05). Next, we examined the possible association between the serrated pancreas and microvascular complications. As shown in Fig. 3 (upper panel). advanced diabetic retinopathy was significantly more common in subjects with serrated changes compared to those without serrated changes (*p* < 0.05). Similar results were obtained regarding diabetic nephropathy. As shown in Fig. 3 (lower panel), advanced diabetic nephropathy was significantly more common in subjects with serrated changes (*p* < 0.005).

## Discussion

Insulin resistance induced by increased visceral fat and fatty liver often contributes to the pathogenesis in type 2 diabetes mellitus^8^. In the previous study using abdominal MRI, the fat content of the pancreas was 9.7% in subjects with normal glucose tolerance and 20.4% in those with diabetes mellitus, and there was also a correlation between the fatty pancreas and HOMA-IR^9^. The increased incidence of type 2 diabetes in subjects with the fatty pancreas also suggests that the fatty pancreas may reflect insulin resistance^10^. In this study, we measured and analyzed pancreatic volume using T1-weighted fat-suppressed MRI, focusing on the decrease in pancreatic volume and serrated changes as findings reflecting an increase in pancreatic fat content. Furthermore, we examined the possible association between morphological changes in the pancreas and vascular complications in subjects with type 2 diabetes and found that morphological changes in the pancreas such as pancreatic volume or serrated changes in the pancreatic limbus were closely associated with vascular complications such as atherosclerosis and diabetic microangiopathy.

A similar study in the past reported a significant correlation between pancreatic volume reduction and HOMA-β^5^. In the same paper, HOMA-β may not have been accurately assessed because the mean blood glucose level at the time of evaluation was above 140 mg/dL^11^. The mean HbA1c of the current participants in this study was 8.9 ± 2.3%, and the analysis was performed using HOMA2-β, which can more accurately assess endogenous insulin secretory capacity even under hyperglycemic conditions^12^. Results showed a significant single correlation with BSA-corrected pancreatic volume, as in previous reports, but no correlation was found in multiple regression analysis. Fatty infiltration into the pancreas leads to decreased β-cell function and has been reported as one factor in the development of diabetes^8^. On the other hand, islets, which are responsible for the endocrine function of the pancreas, account for only about 1% of the volume of the pancreas, and further studies are needed to determine whether a decrease in the overall volume of the pancreas or serrated changes in the pancreatic limbus is directly correlated with the endogenous insulin secretory capacity.

We assume that possible mechanisms for atrophy of pancreatic volume due to fatty pancreas include fat deposition around the adenocellular cells and infiltration from deposited fat, and replacement by fat cells after destruction of adenocellular cells due to microischemia caused by inflammation or atherosclerosis^13^. A 25% increase in pancreatic fat content is associated with the development of type 2 diabetes and atherosclerosis^14^, and fat deposition in the pancreas in subjects with type 2 diabetes leads to insulin resistance and β-cell dysfunction^15^. In the present study, serrated changes were significantly correlated with increased visceral fat area, IMT thickening, and renal dysfunction, suggesting that pancreatic morphological changes in type 2 diabetes may more sensitively reflect ectopic fat deposition in the whole body. Progression of diabetic nephropathy and diabetic retinopathy were also more frequently observed in subjects with type 2 diabetes together with serrated changes. We assume that lipotoxicity, at least in part, contributes to the progression of diabetic microvascular complications in subjects with serrated changes by causing vascular endothelial damage and inducing oxidative stress and apoptosis in extravascular organs. Systemic lipotoxicity should be assumed in subjects with fat deposition in the pancreas, and serrated changes in the pancreas may be a useful finding in estimating the potential risk of microvascular and macrovascular complications of type 2 diabetes mellitus.

This study has several limitations. First, this was a single-center, retrospective study. Most of the patients in this study are Japanese, and their physiques and lifestyles may differ from those in other countries. Second, the number of cases in the control group, non-diabetic patients, was small because the study included patients who had undergone MRI. Larger case numbers are needed for objective validity about the evaluation of pancreatic morphology in non-diabetic patients. Next, it was not possible in this study to determine which factors, such as chronic inflammation, excessive alcohol, or fat deposition, caused the morphological changes in the pancreas. Lastly, it cannot be ruled out that most of the participants in this study had a history of dyslipidemia, potentially influencing the results.

Taken together, decreased BSA-corrected pancreatic volume and serrated changes in the pancreas are closely associated with diabetic microvascular and macrovascular complications, and these findings may be new indicators to reflect ectopic fat deposition in the whole body and to infer the progression of microvascular complications and atherosclerotic changes in subjects with type 2 diabetes mellitus.

## Data Availability

All data generated or analysed during this study are included in this published article.
